# Association between Health and Wealth among Kenyan Adults with Hypertension

**DOI:** 10.5539/gjhs.v13n4p86

**Published:** 2021-03-05

**Authors:** Daniel R. Hanna, Jennifer A. Campbell, Rebekah J. Walker, Aprill Z. Dawson, Leonard E. Egede

**Affiliations:** 1 Center for Advancing Population Science, Medical College of Wisconsin, Milwaukee, WI, United States; 2 Division of General Internal Medicine, Department of Medicine, Medical College of Wisconsin, Milwaukee, WI, United States

**Keywords:** hypertension, wealth index, Kenya, global

## Abstract

**Background:**

This paper examines the relationship between hypertension and wealth in a national sample of Kenyan adults.

**Methods:**

Data from 27,552 individuals from the Demographic and Health Survey Program (DHS) for Kenya were analyzed. Wealth index, a cumulative measure of household standard of living, was the outcome. The final analysis was stratified by gender with covariates added in blocks (demographics, economic, and cultural) to investigate the independent association of hypertension with wealth index.

**Results:**

Approximately 7.6% of those with hypertension had a wealth index above the median. For women and men, hypertension was significantly associated with higher wealth index (women ß=0.26; CI=0.19; 0.34; men ß=0.36; CI=0.19; 0.53). After adjusting for age, rural location, children, employment, education, ethnicity, and religion, hypertension maintained statistical significance with wealth index for both women and men (women ß=0.06; CI=0.01; 0.11; men ß=0.20; CI=0.08; 0.31).

**Conclusions:**

As Kenya as a nation undergoes health care reform while also experiencing a high burden of hypertension, the results presented here provide preliminary evidence that may be used in support for decision makers for the wealth effects of health interventions. Additional work is needed to understand the longitudinal relationship between hypertension and wealth at the national level.

## 1. Introduction

Wealth, characterized as the “value of the assets of worth owned by a person or community, or country”, (Silver, Chen, Kagan, & Woolsey, 2018; Pfeffer & Schoeni, 2016) has been shown to have a strong impact on health outcomes across the world (Wagstaff, 2002). Often referred to as the wealth-health gradient, higher country level and individual level wealth, are shown to be associated with better health at the population and individual level (Semyonov, Lewin-Epstein, & Maskileyson, 2013). While this wealth-health gradient has been demonstrated across countries and population sectors, evidence suggests a bidirectional relationship (Evans, Wolfe, & Adler, 2012). Specifically, wealth, while often serving as a causal factor in health status over a life course, may also be an outcome, mutable to health status (Evans et al., 2012). For example, wealth may decrease as a function of change in health status due to physical inability to work or missed workdays as a result of morbidity and medical appointments (WHO, 2010; Thompson & Conley, 2016; Suhrcke, Nugent, Stuckler, & Rocco, 2006), and associated health related expenditures (Evans et al., 2012; Suhrcke et al., 2005).

Across low- and middle-income countries, this bidirectional relationship between wealth and health may lend to country level health inequities, increasing high poverty rates across sectors as populations with wealth experience a change in health status and deplete resources to improve health (Kazungu & Barasa, 2017). For example, in Kenya it is estimated that less than 20% of the population has health insurance (Kazungu & Barasa, 2017). This suggests that approximately 80% of the population pay for health expenditures out of pocket, resulting in an estimated 400,000 Kenyan adults transitioning to poverty each year as a result of high out of pocket health care expenditures (Kazungu & Barasa, 2017). Evidence shows that these high out of pocket expenditures are often related to managing chronic diseases, such as hypertension (Oyando et al., 2019).

While Kenya is actively engaged in health care reform to increase health insurance across the population (Vision, 2007), the burden of hypertension continues to grow with an estimated prevalence of 24%, exerting severe impact at the national level through individual and economic cost (Kazungu & Barasa, 2017; Oyando et al., 2019). As such, there is an urgent need to understand the impact of hypertension on wealth, as poverty rates are increasing rapidly, and decision makers need information to inform health care reform and resource allocation at the national level (Kazungu & Barasa, 2017). Therefore, the purpose of this paper is to examine the relationship between hypertension and wealth among Kenyan adults using a national sample from the Demographic Health Survey in Kenya. We hypothesized that hypertension would be independently associated with wealth index.

## 2. Methods

### 2.1 Data Source and Study Population

The Demographic and Health Survey Program (DHS) began in 1984 with the goal of collecting nationally representative information to inform policy, plan programs, and understand health, specifically in developing countries (DHS, 2019). The program has conducted surveys in over 90 countries with a wide range of topics including indicators in population, health and nutrition (DHS, 2019). DHS collects data at both the household and individual level to gather information on the topics of interest (DHS, 2019). This study uses data from the household, women’s and men’s questionnaire for the year 2014 in Kenya, the most recently available year of data. All participants who had information regarding hypertension were eligible to be included in the study. In total, there were 27,552 participants included in the analysis.

### 2.2 Wealth Index

The wealth index, included in the household questionnaire, was calculated by DHS and served as the outcome of interest. The wealth index is a cumulative measure of a household’s standard of living (DHS, 2019). The measure incorporates information on household assets, home construction materials and types of facilities available for use (DHS, 2019). Wealth index was used as a continuous variable with higher numbers indicating higher levels of individual wealth.

### 2.3 Hypertension

Hypertension was the primary independent variable. Hypertension was self-reported, and measured through the question “Have you ever been told by a doctor or health worker that you have raised blood pressure or hypertension?” (DHS, 2019) The response options were yes and no; all other values were treated as missing.

### 2.4 Covariates

Possible confounders for the relationship between hypertension and wealth were grouped into demographic, economic, and cultural information. The demographic information used was the respondent’s gender (male, female), age (continuous), location of residence (urban, rural), and the number of children the respondent has (continuous). The economic factors included whether the respondent is currently working (no, yes) and his/her education level (no education, any education). Lastly, ethnicity (Kalenjin, Kamba, Kikuya, Kisii, Luhya, Luo, Meru, Mijikenda/Swahili, Somali, other ethnicity) and religion (no religion, Roman Catholic, Protestant/other Christian, Muslim, other religion) were included as cultural factors.

### 2.5 Statistical Analysis

Sample characteristics were calculated for the overall sample and for those with and without hypertension. The prevalence of hypertension was found for those above and below the median wealth index, for those above and below the age of 45, for each gender, and for urban vs. rural residence. An interaction term was tested to determine if a differential relationship existed by gender. As the interaction term was significant, the final analysis was stratified by gender and was performed using a hierarchical linear regression approach. For each gender, a series of four models were built adding covariates in blocks with hypertension as the primary independent variable in all models. The first model was the unadjusted model with hypertension as the primary independent variable and wealth index as the outcome. The first block added demographics (age, location of residence, number of children), the second block added economic variables (employment and education) and the third block added cultural variables (ethnicity and religion). Stata version 13 (StataCorp, College Station, TX) was used for all analyses with significance being evaluated at the p<0.05 level. To account for complex survey design with clusters, stratification, and weighting, survey commands were used.

## 3. Results

Table 1 shows sample demographics for the overall sample and by hypertension diagnosis. Mean age of the overall sample was 29 years old, and for those with hypertension, mean age was 34 years. While women represented about half of the overall sample, about 75% of those with hypertension were women. In the overall sample, 70% were employed and 95% had some education.

Table 2 shows the prevalence of hypertension by median wealth index, age, sex, and area of residence. Approximately 7.6% of those with a wealth index above the median had hypertension, compared to 5.0% of those with wealth index below the median. By gender, 9.3% of women had hypertension, compared to 3.4% of men.

Tables 3 and 4 show the unadjusted and adjusted hierarchical models for the association between wealth index and hypertension by gender. Table 3 shows in the unadjusted models, hypertension was significantly associated with higher wealth index (ß=0.26; CI=0.19; 0.34). After adjusting for age, location, and number of children, hypertension remained significantly associated with higher wealth index (ß=0.10; CI=0.05; 0.15). Similarly, after adjusting for age, location, number of children, employment, and education, hypertension remained significantly associated with higher wealth index (ß=0.08; CI=0.03; 0.13). Finally, in the fully adjusted model, hypertension was still significantly associated with higher wealth index in women (ß=0.06; CI=0.01; 0.11), though the strength of the association had decreased dramatically.

Table 4 shows the unadjusted and adjusted models for the association between wealth index and hypertension among men. In the unadjusted model, hypertension was associated with higher wealth index (ß=0.36; CI=0.19; 0.53). After adjusting for age, location, and number of children, hypertension remained significantly associated with higher wealth index (ß=0.18; CI=0.06; 0.31). Similarly, after adjusting for age, location, number of children, employment, and education hypertension remained significantly associated with higher wealth index (ß=0.18; CI=0.05; 0.30). In the final model, after adjusting for age, location, number of children, employment, education, and religion, hypertension remained significantly associated with higher wealth index in men (ß=0.20; CI=0.08; 0.31).

## 4. Discussion

Using a representative population sample from Kenya, this study found that hypertension was significantly associated with higher wealth index after adjusting for demographic, economic, and cultural factors for both men and women. While the strength of the relationship decreased for both men and women after adjustment, hypertension remained significantly associated with higher wealth index in both men and women. Additionally, this study found hypertension was more prevalent in women than in men (9.3% compared to 3.4%).

Given the rise in hypertension across Kenya and the subsequent impact on increasing poverty at the individual level due to high out of pocket coverage, (Oyando et al., 2019) this study adds to the evidence base in two important ways. First, from a population level, and consistent with the literature, health as measured by hypertension is associated with wealth. Existing evidence shows that wealth index is associated with risk of hypertension in Kenya (Olack et al., 2015). Specifically, Olack et al. (2015) examined factors associated with increasing rates of hypertension in lower income sectors within Nairobi, Kenya and found that individuals with a higher wealth index had increased risk of hypertension compared to their counter parts with lower wealth index (Olack et al., 2015). While this analysis shows the cross-sectional relationship between hypertension and wealth, these findings support the bidirectional relationship between wealth and health based on existing literature, and that this relationship continues to hold after adjustment for demographic, economic and cultural factors.

Secondly, this study shows that hypertension is associated with wealth in both men and women, and hypertension, while higher among women, is more strongly associated with wealth among men in Kenya. Existing evidence suggests that men and women in Kenya may experience hypertension differentially (Olack et al., 2015; Mkuu, Gilreath, Wekullo, Reyes, & Harvey, 2019; Mohamed et al., 2018, Ploubidis et al., 2013). There is conflicting results around whether men or women have higher prevalence of hypertension, with some studies suggesting women have a higher prevalence, (Gómez-Olivé et al., 2017) while others suggest men have a higher prevalence (Mohamed et al., 2018). Other evidence has also shown that men in Kenya are also more likely to be pre-hypertensive compared to women (Mecha et al., 2020). While the current findings show that women have higher rates of hypertension compared to men, as hypertension was more strongly associated with wealth in men, prevention and intervention efforts should place equal emphasis across men and women and future research should seek to understand how hypertension may decrease wealth across gender, allowing for more targeted intervention efforts. Additionally, as the current study used a self-reported measure of hypertension, awareness of hypertension may differ by gender and future studies should investigate this relationship using a clinical measure of hypertension.

Overall, these findings provide important evidence that can be used at the clinical, research, and policy level to improve population health. Specifically, from a clinical standpoint, existing health care quality for hypertension in Kenya has been impacted by a lack of infrastructure support (Ogola et al., 2019). The Healthy Heart Africa (HHA) initiative was developed to address barriers to hypertension management across a number of counties that target provider knowledge, clinic equipment for screening and management, free hypertension medication, and community education on hypertension (Ogola et al., 2019). The results of the current study support the need for ongoing clinical capacity building as well as highlight the need for hypertension management across men and women in Kenya. Ogola et al. (2019) completed a 12-month evaluation on the HHA in Kenya, highlighting the need for providers to understand the impact that untreated hypertension may have on patient outcomes, of which may need to include economic consequences at the individual level as protocols for treatment are refined and implemented across clinics for screening and treatment of hypertension (Ogola et al., 2019).

From a research standpoint, these results demonstrate a need for population surveillance in Kenya using clinical measures of hypertension and to understand if the relationship with wealth varies across treated versus untreated hypertension. Additionally, there is a need to understand which factors explain the relationship between wealth index and hypertension as a way of identifying targets for future intervention development. An alternative focal point is the role of health insurance in this relationship and to understand if overtime there is a negative impact on wealth, and whether this varies by gender. Specifically, the present findings represent a cross-sectional association between hypertension and wealth. Evidence shows that overall, populations with higher wealth index have more access to medical treatment and therefore may be more likely to be diagnosed with hypertension. While this is one of the first analyses to examine the relationship between hypertension and wealth index as an outcome, further research is needed to understand this relationship from a longitudinal standpoint. Specifically, there is a need to understand how wealth may change among men and women with hypertension and to further understand the mechanisms of this relationship.

At a policy level, the current findings can be used by decision makers as health care reform continues at the national level to support economic stability through population health. Specifically, at the policy level the bidirectional relationship between wealth and health has implications for human capital, the intangible yet integral economically productive aspects of individuals (Becker, 1993; World Bank, 2018; Egede, Walker, Campbell, Dawson, & Davidson, 2020; Cutler, Lleras-Muney, & Vogl, 2010) and has given rise to global perspectives on the wealth effects of health interventions (Garau, Shah, Sharma, & Towse, 2015). Wealth effects, characterized by Garau et al. (2015), are the larger societal benefits of health interventions such as increased productivity and lower health expenditures that result in increased economic capability at the national level (Figueras & McKee, 2012; Figueras, McKee, Lessof, Duran, & Menabde, 2008; Suhrcke, McKee, Sauto, Tsolova, & Mortensen, 2005; Claxton, Walker, Palmer, & Sculpher, 2010). The wealth effects of health interventions may stimulate economic growth by targeting population health (Garau et al., 2015). For example, evidence shows that the economic impact of hypertension in Kenya is extensive, as an estimated 80% of population are without health insurance and pay for medical expenditures out of pocket for such conditions. Additionally, as out of pocket expenditures persist, the annual transition of individuals into poverty may result in devastating effects at the individual and larger societal level over time. Development of health interventions that target hypertension while also implementing health care reform may have a dual impact on the ability of individuals to increase productivity as a result of improved health status while also increasing economic capability through individual productivity across population sectors.

## 5. Limitations

While this study is strengthened by its large sample size and weighting to the population level in Kenya, there are some limitations that need to be considered. First, hypertension was self-reported and may be underrepresented if a large segment of the population is undiagnosed. Second, this analysis is cross-sectional and cannot speak to any causal relationship between hypertension and wealth. Future work should consider clinical measures of hypertension and examine this relationship over time. Finally, while a number of possible confounders were accounted for in this analysis, additional factors, such as health insurance status, were not available in the dataset and may explain some of the relationship between hypertension and wealth index.

## 6. Conclusions

Overall, this study demonstrates a significant relationship between hypertension and wealth index in both men and women using a national sample of adults in Kenya. While the prevalence of hypertension was higher in women compared to men, the relationship between hypertension and wealth index was stronger in men after adjusting for relevant confounders. As Kenya as a nation undergoes health care reform while also experiencing a high burden of hypertension, the results presented here provide preliminary evidence that may be used in support for decision makers for the wealth effects of health interventions.

## Figures and Tables

**Table 1.** Sample Demographics for Overall Sample and by Hypertension

	Overall Sample (N=27,437)	Adults with Hypertension (N= 1,806)	Adults with No Hypertension (N= 25,630)
Characteristics	% or Mean (s.d.)	% or Mean (s.d.)	% or Mean (s.d.)
Wealth Index	0.30 (0.01)	0.58 (0.04)	0.28 (0.19)
Age	29.4 (0.08)	34.2 (0.32)	29.0 (0.09)
Children	2.3 (0.02)	3.1 (0.08)	2.2 (0.27)
Sex			
Male	46.7	24.2	48.3
Female	53.3	75.7	51.7
Region			
Urban	41.9	51.5	41.1
Rural	58.1	48.4	58.8
Employed	70.7	77.9	70.1
Education			
No Education	5.1	4.4	5.1
Any Education	94.9	95.5	94.8
Ethnicity			
Kalenjin	12.08	8.7	12.3
Kamba	11.84	10.2	11.9
Kikuya	21.40	27.0	21.0
Kisii	5.95	4.8	6.0
Luhya	15.85	15.4	15.8
Luo	10.68	12.8	10.5
Meru	5.80	6.5	5.7
Mijikenda/Swahili	5.21	5.6	5.1
Somali	2.29	1.3	2.3
Other	8.89	7.0	9.0
Religion			
Roman Catholic	20.65	23.1	20.4
Protestant/Other Christian	69.74	68.8	69.8
Muslim	6.39	5.7	6.4
No religion	2.80	1.4	2.9
Other	0.41	0.8	0.3

**Table 2.** Prevalence of HTN by Median Wealth Index and Demographics

Characteristics	Percent
Wealth Index	
>=Median WI	7.6
< Median WI	5.0
Age	
>= Age 45	12.0
< Age 45	6.0
Sex	
Male	3.4
Female	9.3
Region	
Urban	8.1
Rural	5.4

**Table 3.** Unadjusted and adjusted linear regression models for the association between wealth index and independent variables among women

Variable	Female							
	Unadjust Bivariate Analysis		Model 1		Model 2		Model 3	
	β	CI	β	CI	β	CI	β	CI
HTN	0.26***	0.19; 0.34	0.10***	0.05; 0.15	0.08**	0.03; 0.13	0.06*	0.01; 0.11
Age			0.03***	0.02; 0.03	0.02***	0.02; 0.02	0.02***	0.01; 0.02
Rural			−1.10***	−1.17; −1.02	−1.07***	−1.14; −0.99	−1.03***	−1.10; −0.96
Children			−0.15***	−0.16; −0.14	−0.13***	−0.14; −0.11	−0.11***	−0.12; −0.10
Working					0.10***	0.06; 0.14	0.09***	0.05; 0.13
Has any Education					0.83***	0.76; 0.89	0.72***	0.64; 0.80
Ethnicity								
Kalenjin							0.03	−0.06; 0.14
Kamba							0.04	−0.06; 0.15
Kikuya							0.42***	0.32; 0.53
Kisii							0.17**	0.04; 0.31
Luhya							0.14**	0.05; 0.24
Luo							0.12*	0.01; 0.23
Meru							0.21***	0.10; 0.32
Mijikenda/Swahili							−0.00	−0.11; 0.10
Somali							0.01	−0.17; 0.19
Religion								
Roman Catholic							0.30***	0.19; 0.41
Protestant/Other Christian							0.27***	0.16; 0.38
Muslim							0.33***	0.18; 0.48
Other							0.89*	0.18; 1.61

Note.

* =p<0.05;

** p<0.01;

*** p<0.001.

Reference groups: Urban, Not Working, No Education, No Religion.

**Table 4.** Unadjusted and adjusted linear regression models for the association between wealth index and independent variables among men

Variable	Male							
	Unadjusted Bivariate Analysis		Model 1		Model 2		Model 3	
	β	CI	β	CI	β	CI	β	CI
HTN	0.36***	0.19; 0.53	0.18**	0.06; 0.31	0.18**	0.05; 0.30	0.20**	0.08; 0.31
Age			0.01***	0.01; 0.02	0.01***	0.01; 0.02	0.01***	0.01; 0.01
Rural			−0.97***	−1.04; −0.90	−0.94***	−1.01; −0.87	−0.93***	−1.00; −.086
Children			−0.07***	−0.10; −0.05	−0.06***	−0.09; −0.04	−0.05***	−0.07; −0.04
Working					0.04	−0.01; 0.10	0.03	−0.03; 0.08
Has any Education					0.92***	0.82; 1.02	0.70***	0.60; 0.81
Ethnicity								
Kalenjin							0.15**	0.05; 0.25
Kamba							0.11*	0.01; 0.21
Kikuya							0.48***	0.38; 0.58
Kisii							0.32***	0.20; 0.44
Luhya							0.19***	0.10; 0.29
Luo							0.14**	0.04; 0.25
Meru							0.28***	0.17; 0.38
Mijikenda/Swahili							−0.05	−0.16; 0.05
Somali							−0.20*	−0.38; −0.02
Religion								
Roman Catholic							0.30***	0.20; 0.40
Protestant/Other Christian							0.31***	0.22; 0.40
Muslim							0.35***	0.19; 0.51
Other							0.33	−0.04; 0.71

Note.

* =p<0.05;

** p<0.01;

*** p<0.001.

Reference groups: Urban, Not Working, No Education, No Religion.
